# Supplementary material for: Calcium supplementation in pregnancy: An analysis of potential determinants in an under-resourced setting
Source: PLoS One. 2023 Oct 5;18(10):e0292303. doi: 10.1371/journal.pone.0292303 (PMC10553325; doi:10.1371/journal.pone.0292303)
Supplement: Attachment — Submitted filename: Response to Reviewers.docx [file pone.0292303.s002.docx]

**Response to reviewer’s comments**

| **Editor’s comments** | **Responses** |
| --- | --- |
| Please revise this manuscript point by point as per the review comments. I also suggest the authors should address the research questions presented in the registered report protocol and should support the conclusions by the data. | Thank you for your recommendation. We presented a point by point response to reviewers on this table. This manuscript is presented just for objective 2 of the objectives presented on the registered protocol. Other questions raised on this protocol have been published (1) and others are under review (PONE-D-21-33966R3). We have tried to answer all reviewers’ comments and hope our conclusions are in line with the objectives of this manuscript. |
| Please ensure that your manuscript meets PLOS ONE's style requirements, including those for file naming. | Our manuscript has been formatted to respect PLOS One guidelines. The files have been named accordingly. |
| We note that you have stated that you will provide repository information for your data at acceptance. Should your manuscript be accepted for publication, we will hold it until you provide the relevant accession numbers or DOIs necessary to access your data. If you wish to make changes to your Data Availability statement, please describe these changes in your cover letter and we will update your Data Availability statement to reflect the information you provide. | Thanks for suggestion. We have now changed our data availability statement to “All relevant data are within the paper and its Supporting information file”. The data base has been submitted with this revision as a supporting information file. |
| Please ensure that you refer to Figure 1 and 2 in your text as, if accepted, production will need this reference to link the reader to the figure. | These two figures have been mentioned in the text. See line 258-264. |
| **Reviewer’s comments** | **Responses** |
| Thank you for the opportunity to review this paper about factors that influence calcium supplementation in Nkongsamba Cameroon. Calcium supplementation has been recommended by WHO for years but there are few countries where it is included as part of ANC and there is still limited research about the factors that influence adherence. This is an important study to help understand more about factors that influence Ca supplementation. However, this paper can be strengthened by providing more information about the context/setting and a clearer definition of adherence measures. My comments are below: | Thank you for your assessment. We greatly appreciate the time and efforts put in to extensively review this manuscript and recommend the changes. We must agree that this has gone a long way in strengthening and bringing more sense to this piece of work. We have addressed each of your comments and provided explanations and amendments to the manuscript. We hope that our efforts meet you expectations. |
| Overall More information about ANC in Cameroon is needed to provide context for this paper. Is Ca supplementation recommended by the Ministry of Health? Are Ca supplements provided for free as part of ANC? (it sounds like women are purchasing Ca supplements which is surprising, how does this differ from IFA is that provided for free or are women used to purchasing their own supplements). How many supplements are recommended and provided? Is there information about the types of Ca supplements that are provided (mg)? How do women know how many mg they are taking? What are the rates of preeclampsia/hypertensive disorders in the country? How many ANC visits are recommended by the MOH? How many ANC visits do most women make based on the authors' previous, DHS, or other data? What about other micronutrient supplementation programs in the country as part of ANC. Is IFA recommended, multiple micronutrient supplements? Has Ca supplementation been promoted? Have ANC providers been trained on Ca supplementation? Is there data about dietary Ca intake in Cameroon and/or Nkongsamba. | Thanks for the suggestions. The whole introduction has been reviewed as recommended. Please see the introduction, page 4-7. |
| A section on measures needs to be added to the methods. Each calcium supplementation measure needs to be defined and the process or collecting this information and creating categories needs to be described. The use of the word prevalence is unclear as it is used throughout this paper. Noting that the interview guide is available in the protocol paper would help readers who want more detail. But a short summary is also needed. | Thank you for the recommendation. This information has been included. See paragraph 2, page 10.  The interview guide has also been cited in the methods. See line 194, paragraph 1, page 10 |
| How do the findings from this study compare with IFA supplement adherence in the area? Does it seem consistent? Do women in Nkongsamba typically purchase IFA supplements in pregnancy? | We have now presented data on the IFA supplementation on table 2. See last paragraph page 16 and page 17.  As stated in the background, all supplements in pregnancy as purchased by the client. |
| These data were collected during COVID-19, how did that impact access to ANC, calcium supplies, etc? | Thanks for the question. We have no study around the hospital which was aimed at evaluating the impact of COVID-19 ANC access. However, we belief that COVID-19 might have reduce the number of ANC contacts for some women. But given the fact our data was collected in late pregnancy, we think that most women even if did not have a complete ANC follow-up, they would have shown up in late pregnancy. All supplements during ANC are prescribed and are bought by the client. We don’t have information or data on how COVID-19 might have affected calcium supplies in the pharmacies. |
| In the conclusion, the authors highlight the need to involve male partners. Are other family members such as grandmothers (pregnant women's mothers and mothers-in-law) also influential during this time? Would it potentially be important to involve them as well? | Thank you for your question. This has been included as a limit of our study given that we failed to collect data on the support of other groups and family members. See last paragraph of discussion, line 419 |
| The following paper may be helpful for framing the importance of this research Gomes F, Ashorn P, Askari S, Belizan JM, Boy E, Cormick G, Dickin KL, Driller‐Colangelo AR, Fawzi W, Hofmeyr GJ, Humphrey J. Calcium supplementation for the prevention of hypertensive disorders of pregnancy: current evidence and programmatic considerations. Annals of the New York Academy of Sciences. 2022 Jan 8. | Thank you very much for your extensive research and review. This article was of great help to us. We have added it to our references. |
| **Specific comments reviewer’s comments** | **Responses** |
| Title – consider another term than low-resource setting. Could either name the area Nkongsamba or Cameroon or use under-resourced setting | Thank you for your suggestion. Under-resourced setting has been used in the title. See title |
| Introduction Lines 61-68 - I suggest starting the introduction with the second paragraph about the health consequences of low calcium intake to help the reader understand why Ca supplementation is recommended, and then the paragraph with information about prevalence of low calcium in subsequent paragraphs | Thanks for your suggestion. The whole introduction structure has been reviewed and we tried to organise ideas as recommended. See introduction. |
| Line 69 – use of the word refined here is not clear, suggest deleting | Thanks very much. We have now taken off the word refined. See line 94. |
| Line 71 – While the protocol focuses on serum Ca levels this seems less relevant for this paper | Thank you for your comment. The statement was just to emphasise the consequence of low calcium intake on calcaemic states from recent reviews, thereby indicating the need for calcium supplementation. |
| Line 75 – clarify this is pregnant women | Thank you for the suggestion. The word “pregnant” has been added. See line 123. |
| Line 82 – is this pregnant women in China, please clarify.  Line 82 – should this be affect calcium supplementation “adherence”? | Thank you for the question. This is in pregnancy (China and Bangladesh). The word “pregnant” has been added. See line 114.  Thanks for the request of specificity. Actually these factors affected adherence. See line 123. |
|  |  |
| Line 83 – food insecurity/household hunger has been associated with poorer adherence to Ca supplements in Kenya (see the following article : Martin SL, Omotayo MO, et al. Adherence partners are an acceptable behaviour change strategy to support calcium and iron‐folic acid supplementation among pregnant women in Ethiopia and Kenya. Maternal & Child Nutrition. 2017 Jul;13(3):e12331.) | Thanks for the information. This information has been included in the background section. See line 125-127. |
| Line 86-87 – Please refer to papers by Omotayo et al describing a study on Ca supplementation in Kenya  Omotayo MO, Dickin KL, Pelletier DL, Martin SL, Kung'u JK, Stoltzfus RJ. Feasibility of integrating calcium and iron–folate supplementation to prevent preeclampsia and anemia in pregnancy in primary healthcare facilities in Kenya. Maternal & child nutrition. 2018 Feb;14:e12437.  Omotayo MO, Dickin KL, Pelletier DL, Mwanga EO, Kung'u JK, Stoltzfus RJ. A simplified regimen compared with WHO guidelines decreases antenatal calcium supplement intake for prevention of preeclampsia in a cluster-randomized noninferiority trial in rural Kenya. The Journal of nutrition. 2017 Oct 1;147(10):1986-91. | Thank you very much for these references, they have been used in the background. See line 125-135. |
| Line 88 - Please include a reference for this study about prevalence? | Thank you for your suggestion. The reference has been added. See line 141. |
| Line 88 – what is meant by supplementation prevalence? Please clarify if it is pregnant women taking any Ca, taking recommended amount of Ca (and stating what that amount is)? Something else? | Thank you for the question. The precision has been made throughout the manuscript. The “prevalence” has been taken of. The proportion of women who report to have taken calcium supplements (any form, dose and duration) in pregnancy is used. It has also been defined in the methods. See 197-204. |
| Line 90 – is obstetric the correct word here? Is it related to child birth or pregnancy? I believe it is pregnancy. | Thanks for the suggestion. The word has been changed to “pregnancy-related”. See line 143. |
| Line 95 - clarify that this is referring to a protocol | Thanks very much. This has been made clear. See line 148 |
| Line 95 – clarify that this is a secondary data analysis | Thank you for the suggestion. This manuscript was designed to respond to objective 2 of the registered protocol which intended to identify barriers to calcium supplementation among these women. |
| Line 97 and 116 – was it a structured survey or semi-structured interview guide? Typically I consider a semi-structured interview guide to be used to collect qualitative data and it suggests the interviewer has flexibility in terms of which questions are asked and how - I imagine all questions were meant to be asked in the order they were written? | Thanks for your questions; the questions were not always asked the way they were written on the questionnaire. In some cases and some questions, the meaning of the question was further explained to the client to make sure the information given was adapted to the question asked. |
| Line 102 - Please provide more information about Nkongsamba are all these health facilities in urban areas? Other than NRH what level hospital are the others? | Thank you for the request. The requested information has been provided. See line 157-167. |
| Line 110 – how was sample size determined? | Thank you for your question. Information on the determination of the sample size has been presented. See line 175-180. |
| Line 110 – Please describe attempts that were made to address potential bias. | Thanks very much. A section has been added on bias. See line 205-211. |
| Line 121 – Please note how many participants refused to participate. | Thanks for the request. The non-response rate has been presented in the first paragraph of the result section. See line 239-240. |
| Line 127 – What language were the interviews conducted in? | Thanks for the question. The interview was conducted either in English or French depending on the preference of the client. This is indicated on the manuscript. See line 185-187. |
| Line 127 – How was calcium supplementation measured and defined? How was it asked? I see there are descriptions of daily dose, how was this determined? | Thank you for the question. This has been presented in the methods. See line 197-204. |
| Line 129 – Please describe how missing data were handled | Thanks for the recommendation. This information has been added. See line 215-221. |
| Table 1 and line 134. Is revenue household income or her individual income? Please clarify | Thanks for the question. This referred to her individual monthly income. See table 1. |
| Line 155 – please use married in place of union | Thanks for suggestion. Union is used here to designate legally married women and those who are cohabiting. The precision has been made to the phrase. See line 245-246. |
| Line 158 – having the definition of the Ca measures in the methods would really help this paragraph | Thank you for the suggestion. This has been added to the methods section as requested. |
| Line 161-162, what is meant by all through pregnancy – every day all through pregnancy? Every day since they knew they were pregnant? Is there a recommendation for when calcium supplementation should start in Cameroon? Is it at 20 weeks or before? Is Ca supplementation mentioned in national ANC guidelines? | Thanks for request for the precision. All through pregnancy was used for women who declared that they took calcium supplements from the time they knew they were pregnant. This precision has been included in bracket. See line 261.  Cameroon has no recommendation as to when to initiate calcium supplementation in pregnancy and calcium supplementation in pregnancy is not a standard practice as per the national ANC guidelines. |
| Table 2 – what is the definition of Ca supplementation in this table? | Thanks for the question. The definition of calcium supplementation in the study has now been presented in the methods section and table 2. See page 9, last paragraph and table 2. |
| Line 205 – clarify what is meant by prevalence of calcium supplementation in pregnancy? | Thanks for the question. The prevalence of calcium supplementation in pregnancy has now been defined in the methods section. See page 10, line 197-204 |
| Line 225 – specify this is in populations with low dietary calcium intake (not in LMIC) | Thank you very much for the correction. This correction has been made. See line 350-51 |
| Line 234 – in Kenya with consistent supply, health worker training, family engagement adherence rates were quite high. Consider referencing these findings as well as the low adherence rates in Kenya  Omotayo MO, Dickin KL, Pelletier DL, Mwanga EO, Kung'u JK, Stoltzfus RJ. A simplified regimen compared with WHO guidelines decreases antenatal calcium supplement intake for prevention of preeclampsia in a cluster-randomized noninferiority trial in rural Kenya. The Journal of nutrition. 2017 Oct 1;147(10):1986-91. | Thank you for your suggestion. This information has been included and referenced. See line 360-364. |
| Line 249 – include reference for this statement Line 250 – include reference for this statement | Thanks for the recommendation. These statements have been referenced. See line 379-381. |
| Line 253 – please clarify what is meant by obstetric outcomes, I read it as maternal and infant mortality/birth outcomes but based on the sentence that follows it seems to be adherence to supplements – please clarify. If it is obstetric outcomes please add a reference, if supplement please clarify. | Thanks for the suggestion. Its obstetric outcomes and the references have been added. See line 380. |
| Line 256 – what is meant by antenatal follow up? | Thank you for the question. Antenatal “care” has been used in the place of “follow-up”. See line 387. |
| Line 257 is this supplementation adherence? | Thank you for the question. It has to do with adherence. The precision has been made in the manuscript. See line 388. |
| Line 260 – is this also the recommendation in Cameroon? | Thanks for the question. This WHO recommendation is adopted for Cameroon. The precision has been added to the sentence. See line 390. |
| Line 266 – Consider referring to this paper which focuses on partner support  Martin SL, Omotayo MO, et al. Adherence partners are an acceptable behaviour change strategy to support calcium and iron‐folic acid supplementation among pregnant women in Ethiopia and Kenya. Maternal & Child Nutrition. 2017 Jul;13(3):e12331. | Thank you for the suggestion. This paper has been read and referenced. See line 398. |
| Line 276 include year for reference | Thanks for the request. The year of the reference has been added. See line 407. |
| Line 284 – is this referring to perceived side effects? | Thanks for the question. We meant perceived side effects. See line 418. |
| Line 286 – what about counseling about calcium | Thank you for the addition. This has been integrated into a limit. See line 421-422. |
| Figure 2 – how was daily dose of Ca determined? | Thank you for the question. This information has been provided in the methods section. |
| Thank you for including the interview guide in the protocol paper. Including the results from some of these questions would help provide context for the results 1. Have you been advised by health personnel on the necessity to take calcium supplements? a)Yes b)No 2. Why is it important to take calcium supplements in pregnancy? a) I don’t know b) For foetal bone development and growth c) to prevent cramps in the mother d) prevent high blood pressure in pregnancy, e)others 3. Did you take iron and folic acid supplements in pregnancy? a) Yes b) No , c)I took only folic acid, e)I took only iron supplements. 4. If yes, How often? a) Everyday b) At least once every two days c) Rarely 5. Do you take calcium supplements at the same time as the iron and or folic acid supplements? a) Yes b)No c) I don’t take calcium supplements  Also it would be important to note that partner support question was not specific to calcium supplements but appears to be asking about both IFA and calcium supplements, please clarify this.  6. Does your partner support you and remind you on the need to take your supplements? a) Always b)Sometimes c)Occasionally d) Never | Thank you for your recommendation. We have now included table 2 with analysis of these variables. |

**References**

1.	Ajong AB, Kenfack B, Mbulli Ali I, Yakum MN, Ukaogo PO, Mangala FN, et al. Ionised and total hypocalcaemia in pregnancy: An analysis of prevalence and risk factors in a resource-limited setting, Cameroon. Spradley FT, editor. PLoS One [Internet]. 2022 May 18 [cited 2022 May 26];17(5):e0268643. Available from: https://journals.plos.org/plosone/article?id=10.1371/journal.pone.0268643
