# Supplementary material for: Calcium supplementation in pregnancy: An analysis of potential determinants in an under-resourced setting
Source: PLoS One. 2023 Oct 5;18(10):e0292303. doi: 10.1371/journal.pone.0292303 (PMC10553325; doi:10.1371/journal.pone.0292303)
Supplement: Attachment — Submitted filename: Response to Reviewers.docx [file pone.0292303.s003.docx]

**Response to Reviewers**

| REVIEWER’S COMMENTS | RESPONSE |
| --- | --- |
| Thank you for the opportunity to review this revised manuscript about calcium supplementation in Cameroon. The manuscript has been substantially improved and the information about the contextual information in Cameroon is helpful to understand the setting. All line numbers refer to the tracked changes version of the manuscript. | Thank you very much for taking the time to review our manuscript. We are grateful |
| It is still surprising that health workers are recommending/prescribing calcium supplements since it is not included in the ANC guidelines. It would be helpful if the authors could provide some explanation for why ANC providers are recommending calcium (Lines 110-1112) | Thanks for your observation. Health workers sometimes violate national guidelines especially when growing evidence is showing the benefits of the practice. Some health professionals do prescribe calcium supplements systematically, some prescribe depending on the client’s symptoms and request. Line 103 to 104 has been added to make this clear. |
| It would be helpful if throughout the manuscript any time the authors reference women have taken calcium supplements or womens calcium supplementation that it would be written as have taken any calcium supplements just to make it clear that it is not referencing a specific amount dose or duration. While this is noted in the methods it would be a helpful reminder to the reader if it were throughout the manuscript. | Thank you for your suggestion. We have edited the manuscript to make this clear following your suggestion. |
| Line 35 – please revise proportion of women who reported calcium supplementation to proportion of women who reported [taking any] calcium supplements Line 63-64 please add references | Thank you for the suggestion. It has been corrected. See line 37. Also the reference has been added, see line 65 |
| Line 102 – consider deleting only as this is not such a low number (there are several places where only, just, meager, other words are used, consider reviewing and revising whether or not these are appropriate) | Thanks for your observation. “Only” was used to say that it is far from target. 87% means 13% went through pregnancy without receiving medical care from a qualified health personnel. See line 92-95. I have also reviewed the manuscript to see if the words used are appropriately placed. |
| Line 105 – please add promoted by who - the MOH or in the national ANC guidelines? Line 126 consider revising "depend on" to "influenced by" | Thank you for the suggestions. This corrections has been made. See line 96 and 97 and line 119. |
| Line 129 – add "support" to partner adherence [support] | Thank you for the correction. We have addressed this on the manuscript. See line 123. |
| Line 205 the bias section could be moved to the end of the discussion in a limitations paragraph Line 217, please clarify, what is the study document? | Thanks you for the review. The bias section has also been carried to the end of the discussion as requested. See 421-428.  Also correction has been made to clarify the study document. It is the participant information sheet. See line 209. |
| Line 224 please revise "between calcium supplementation" to "taking any calcium supplements" (and change throughout eg lines 315-315, 324 but many other places as well) | Thank you for this correction. The whole document has been verified accordingly. See line 217. |
| Line 257 – please add "any" – took any calcium supplements | Thanks for the suggestion. This has been added. See line 246. |
| Line 274 – consider deleting the word some Line 279– please add any – without taking any IFA supplements Line 323-339, rather than restating the results could describe women’s understanding of why to take supplements and provide more of an explanation | Thanks for the suggestions. These corrections were adopted. See lines 261, 266, and line 305-314. However, we prefer to have a small reminder of the key findings of the study here. |
| Line 340 – please revise, could consider something like: In our study 72.62 [69.85-75.22]% of participants reported taking any calcium supplements during pregnancy. | Thanks for your suggestion. This was adopted. See lines 331-333. |
| Line 345 – why are prescription practices likely to have changed? | Thank you for the question. We think this might have been caused by a change in the personnel of the department which could have affected practices. |
| Line 355 – please add a reference | Thanks for the request. The reference has been added. See line 349. |
| Line 360-361, mean adherence in these studies was 86%, this is not low, consider revising | Thank you for the correction. The sentence has been reformulated. See lines 351-353. |
| Line 412 add date for Nguyen et al reference | Thanks very much. The date has been added. See line 403. |
| Conclusion – should Cameroon adopt the WHO guidelines about calcium supplementation | Thank you for the question. Our study evaluated factors influencing calcium supplementation. We did not re-evaluate the benefits of calcium supplementation in pregnancy. So based only on the results of this manuscript, we cannot give this recommendation. However, substantial evidence recognises calcium supplementation in pregnancy to have beneficial results for the mother and child. We found out that with respect to WHO standards, calcium supplementation in Cameroon in pregnancy is not carried out adequately. We encourage women to supplement in calcium while fighting to meet most of their calcium needs in the diet. However, we think Cameroon should adopt the WHO recommendations on calcium supplementation in pregnancy. |
| Line 444 please delete “their “from their women’s adherence | Thank you for the suggestion. The word has been deleted. See line 442 |
| Line 444-445 - do your study findings justify a recommendation about involving men in ANC, if so consider adding this recommendation and references in the discussion earlier. There are examples of challenges with involving men in ANC in other settings in Africa and several studies about male involvement in ANC related to PMTCT and not. Are there other ways to involve male partners to support calcium supplementation based on the literature (for example from the study in Bangladesh) that could complement recommendations about involving men in ANC (community mobilization, mass media, home visits) since getting men to ANC visits can be challenging. | Thank you for the question. Our study results suggest that the male partner could contribute to the client’s adherence to prescribed protocols. This indirectly means that men could play a role. We had this information added in the discussion section. See lines 384-390.  Thanks for the question. We have added a few precisions on how men could be involved. See lines 443-444. |
