# Supplementary material for: Calcium supplementation in pregnancy: An analysis of potential determinants in an under-resourced setting
Source: PLoS One. 2023 Oct 5;18(10):e0292303. doi: 10.1371/journal.pone.0292303 (PMC10553325; doi:10.1371/journal.pone.0292303)
Supplement: Attachment — Submitted filename: Response to Reviewers.docx [file pone.0292303.s004.docx]

**RESPONSE TO REVIEWERS**

We appreciate the time taken by the reviewers and editors to fully evaluate and strengthen this manuscript. We have now responded to the comments raised by the reviewer. This is presented here in a tabular form. As mentioned in the cover letter during the last revision, we also have made some changes on the reference list. The correct version of reference 16 has been provided. Also reference 17 has been taken off (it was a retracted version of reference 2). Also reference 26 and 27 have been interchanged.

| Comment | Response |
| --- | --- |
| 1. Becasue the aim of this study is to invetstigate the affecting factors, what potential facors did the authors cared about? And why to select them? Authors should clearly state this informaiton in the part of methods. | For this study, potential predictors of calcium supplementation included age of the participant, her level of education, level of education of partner, household size, gestation age at first visit, number of daily meals, occupation, number of ANC and support from partner. These factors were selected because they were reported I previous study to be associated with some preventive interventions’ uptake. The authors wanted to check the context specificities of this potential association. This has been added in the methods. See data analysis section, line 205-211. |
| 2. In the part of data anlaysis, author said that "the mean (µ) ...." it is wrong expression. For smaple analysis, µ is not apprioriate because it is for population. | Thank you for this comment. We agree with the reviewer, and we have taken this symbol off. see lines 204, 205 and 228. |
| 3. For logisitc regression, it is unclear about depentent variable. A clear definition is reqiured. Moreover, if dependent variable is "the proportion of women who took any calcium supplements during pregnancy", logistic model could not be suitable because the propotion is hiher (72.6%), which could overestimate the odds. Possion regeression is suggested. | Thank you for this comment.   1. We have included clear definition of the outcome variable at the level of methodology of the manuscript. See lines 183-190 and line 212. 2. It is unclear to the authors, the reason the reviewer thinks that higher proportion of outcome variable in the study sample would lead to overestimation of the odds in logistic regression. A more elaborate description on how logistics regression is used can be seen in the book Logistic Regression Models by Joseph M. Helbe (<https://doi.org/10.1201/9781420075779>) equally, a systematic review (<https://www.scirp.org/journal/paperinformation.aspx?paperid=95655°> ) describe the section of outcome variable for logistic regression. In all, the decision to use logistic model is based more on the nature of the outcome variable and not on value of the outcome. Moreover, we endeavored to present our associations with precisions that these were “odds” not “risk” nor likelihood. 3. Besides, Poisson regression can not be applied in this study because it is recommended for studies with count outcomes and not categorical nor continues outcomes (<https://www.tandfonline.com/doi/abs/10.1080/01621459.1987.10478502> ) 4. We believe that Logistic regression remains the best method to make this analysis based on the dichotomic nature of the outcome variable. |
| 4. Due to few covariates considered in this study, all covariates are suggested to be included in the adjusted analysis not just included covariates which are significant at P less than 0.25 in simple logisitc regression. | Thank you very much for this comment. We agree with the reviewer that fewer independent variable in the logistic model may make the model to explain greater or lesser variance than it is in reality. However, we would like to cite a section of this review on logistics regression in medical research (<https://www.scirp.org/journal/paperinformation.aspx?paperid=95655> ) on this issue. It states “Unfortunately, the solution is not simply to include as many variables as possible, as the inclusion of variables that are unrelated to the outcome in question, this (the addition of unrelated variables) has the tendency to inflate the apparent predictive validity of the final model”. Several methods exist for the selection of the variables to be included in the model (doi: [10.1136/fmch-2019-000262](https://doi.org/10.1136%2Ffmch-2019-000262)) and the use of 0.25 p-value cutoff from bivariate analysis aims to reduce the inclusion of unrelated variables |
