# Supplementary material for: Calcium supplementation in pregnancy: An analysis of potential determinants in an under-resourced setting
Source: PLoS One. 2023 Oct 5;18(10):e0292303. doi: 10.1371/journal.pone.0292303 (PMC10553325; doi:10.1371/journal.pone.0292303)
Supplement: Attachment — Submitted filename: RESPONSE TO REVIEWERS.docx [file pone.0292303.s005.docx]

RESPONSE TO REVIEWERS

Reviewer #1: The authors responded to my comments on the previous round of revisions and I do not have any additional comments.

**Response: We really appreciate the time you have put in to review this manuscript for the second time. Your comments have gone a long way to strengthen this manuscript.**

Reviewer #2: Authors have addressed most of my comments. I am still concerned about the following issues:

**Response: We really appreciate the efforts you have put in to strengthen this manuscript and thank you for sending back these comments. We hope that our answers meet your expectations.**

1. It is clear that Logistic model could overestimate effect size if prevalence is greater than 10% (a body of literature have addressed it). Generally this model is for issue of rare event or lower prevalence of event of interest.

**Response: We agree that logistic regression can overestimates effect size when the prevalence of the outcome is high. However, we feel that using logistic regression to bring out the information this study was designed for is not a problem. This is for the following reasons.**

- **Logistic regression and odds ratio is the most valid, if not, the only valid statistical method which is adapted for a cross sectional population study like ours. This method of analysis has been used in uncountable studies with relatively high prevalence of the outcome of interest [1–12].**
- **We feel that the error comes when the odds ratio is confused with the relative risk or prevalence ratio or directly translated to risk. When the results are reported in terms of increasing or reducing odds, the reader understands that in terms of magnitude of risk, it is not the same magnitude. In this particular study we did not intend to quantify the risk. Increasing or decreasing odds also mean increasing or decreasing risk even though not of the same magnitude** [13]**.**
- **Logistic regression overestimates odds ratios in studies with small and moderate sample sizes** [14]**. This is not the case of our study which included more than 1000 women.**
- **The problem of the exact effect size or magnitude of risk is very important only in clinical trials where the magnitude of the odds ratio could directly be used to take decisions. Population studies evaluating behavior-linked practices identify more of factors and their direction of influence which is easy to state from our manuscript. Therefore, we donot really see the statistical method here affecting the outcome of this study.**
- **All reported results have been stated in the manuscript in terms of increasing and decreasing number of odds.**

2. Selection of variable should be based on objective of study and sample size. There was fewer covariates considered in this study with samples size of more than 800 and the aim is to identify the potential variables. It is uncessary to select by uivariate analysis. Authors cited the reference which is just for clinical prediction modelling where logisically limited number of covariates is necessary for prediction.

**Response: Thanks very much for your worries. We belief this is with the objective of strengthening the work. We understand your worries. However, our covariates for potential risk factors were selected based on literature and its plausible potential to have an effect. All variables we felt could intervene were included in the logistic regression model. Some explanations to this had been included in the past reviews in the data analysis section page 11. What we don’t understand is whether there is a maximum number of variables we must have collected data on to be able to apply our model. We are surprised to have this as a problem because from publications we read every day, emphasis is not put on any limit of variables to reach.**

**We also acknowledged in the last paragraph of the discussion (page 24) that we did not collect data on all potential factors which could influence calcium supplementation adherence as a strong limit of our study.**

References

1.	Tesfaye A, Alemayehu M, Abere G, Mekonnen T. Prevalence and Associated Factors of Computer Vision Syndrome Among Academic Staff in the University of Gondar, Northwest Ethiopia: An Institution-Based Cross-Sectional Study. Environ Health Insights. 2022;16: 117863022211118. doi:10.1177/11786302221111865

2.	Tesfaye A, Mekonnen T, Alemayehu M, Abere G. Prevalence and Risk Factors of Work-Related Upper Extremity Disorders among University Teaching Staff in Ethiopia, 2021: An Institution-Based Cross-Sectional Study. Pain Res Manag. 2022;2022: 1–12. doi:10.1155/2022/7744879

3.	Tang L, Lee AH, Yau KKW, Hui YV, Binns CW. Consumption of dietary supplements by Chinese women during pregnancy and postpartum: A prospective cohort study. Matern Child Nutr. 2017. doi:10.1111/mcn.12435

4.	He G, Yang H, Chen M, Liu X. Factors influencing folic acid, multivitamin, and calcium supplementation among pregnant women in China based on a national cross-sectional survey. Chin Med J (Engl). 2023;136: 473. doi:10.1097/CM9.0000000000002202

5.	Adimasu A, Azene Z, Merid M, Goshu A, Geberu D, Kassa G, et al. Knowledge and attitude of the communities towards COVID-19 and associated factors among Gondar City residents, northwest Ethiopia: A community based cross-sectional study. PLOS ONE. 2021;16: e0248821. doi:10.1371/journal.pone.0248821

6.	Wami S, Mekonnen T, Yirdaw G, Abere G. Musculoskeletal problems and associated risk factors among health science students in Ethiopia: a cross-sectional study. J Public Health. 2021;29: 1–7. doi:10.1007/s10389-020-01201-6

7.	Mekonnen T, Tesfaye Y, Moges H, Berhe R. Factors associated with risky driving behaviors for road traffic crashes among professional car drivers in Bahirdar city, northwest Ethiopia, 2016: A cross-sectional study. Environ Health Prev Med. 2019;24. doi:10.1186/s12199-019-0772-1

8.	Geberu D, Biks G, Gebremedhin T, Mekonnen T. Factors of patient satisfaction in adult outpatient departments of private wing and regular services in public hospitals of Addis Ababa, Ethiopia: a comparative cross-sectional study. BMC Health Serv Res. 2019;19. doi:10.1186/s12913-019-4685-x

9.	Mekonnen T. The magnitude and factors associated with work-related back and lower extremity musculoskeletal disorders among barbers in Gondar town, northwest Ethiopia, 2017: A cross-sectional study. PLOS ONE. 2019;14. doi:10.1371/journal.pone.0220035

10.	Utami A, Margawati A, Pramono D, Nugraheni A, Pramudo SG. Determinant Factors of COVID-19 Vaccine Hesitancy Among Adult and Elderly Population in Central Java, Indonesia. Patient Prefer Adherence. 2022;16: 1559–1570. doi:10.2147/PPA.S365663

11.	Berihun G, Walle Z, Teshome D, Berhanu L, Derso M. COVID-19 Vaccine Acceptance and Associated Factors Among College Students in Dessie City, Northeastern Ethiopia. J Multidiscip Healthc. 2022;15: 1735–1746. doi:10.2147/JMDH.S381151

12.	Gedif G, Sisay Y, Alebel A, Belay YA. Level of job satisfaction and associated factors among health care professionals working at University of Gondar Referral Hospital, Northwest Ethiopia: a cross-sectional study. BMC Res Notes. 2018;11: 824. doi:10.1186/s13104-018-3918-0

13.	Barros AJ, Hirakata VN. Alternatives for logistic regression in cross-sectional studies: an empirical comparison of models that directly estimate the prevalence ratio. BMC Med Res Methodol. 2003;3: 21. doi:10.1186/1471-2288-3-21

14.	Nemes S, Jonasson JM, Genell A, Steineck G. Bias in odds ratios by logistic regression modelling and sample size. BMC Med Res Methodol. 2009;9: 56. doi:10.1186/1471-2288-9-56
